# Advancing health equity through simulation-based education for pre-health students

**DOI:** 10.1371/journal.pone.0354377

**Published:** 2026-08-12

**Authors:** Oluwakemi Tomobi, Sarah DeFazio, Dena Lin, Alyssa Brashear, Klint Smart, Gregory Epps, Kerri Woodberry

**Affiliations:** 1 Department of Anesthesiology, West Virginia University, Morgantown, West Virginia, United States of America; 2 Department of Psychiatry, West Virginia University, Morgantown, West Virginia, United States of America; 3 West Virginia University, Morgantown, West Virginia, United States of America; 4 West Virginia University School of Medicine, Morgantown, West Virginia, United States of America; 5 Health Sciences & Technology Academy, West Virginia University, Morgantown, West Virginia, United States of America; 6 Department of Surgery, West Virginia University, Morgantown, West Virginia, United States of America; University of Porto Faculty of Medicine: Universidade do Porto Faculdade de Medicina, PORTUGAL

## Abstract

**Introduction:**

Basic sciences are foundational for a career in the health professions. However, interest in the sciences wanes as students in the United States progress through K-12 education and reaches a nadir in the undergraduate years; this decline is particularly marked in underrepresented communities. In addition, difficulty in basic sciences may present as a barrier to entering the health professions. This study examines clinical simulation as an adjunct to lectures for promoting pre-health students’ understanding, retention, and recall of the basic sciences as well as the feasibility and acceptability of this approach.

**Methods:**

This study used a pretest/posttest design. Data collection occurred from March 2025 to August 2025. Following a traditional lecture, simulation was conducted on the following scenarios: malignant hyperthermia, opioid overdose, stroke, and cardiac emergency. Students assumed different roles within a healthcare team, allowing for checklist assessment of knowledge acquisition and decision-making abilities in a controlled setting. Written 20 item pre-simulation and post-simulation assessment data were analyzed descriptively and with t-tests to assess learning. Surveys and qualitative analysis from oral and written comments were used to assess acceptability and feasibility of simulation as an educational tool.

**Results:**

Clinical simulation participants included 43 pre-health students. There was a mean increase in written test scores following simulation, demonstrating significantly higher performance immediately after the simulation and 3 months later (*M*1 = 10.4 vs *M*2 = 16.4; *p* < 0.001; M1 = 10.4 vs M3 = 16.2, p < 0.001). Participants reported increased confidence (p < 0.001), most significantly in understanding stroke, heart attack, intraoperative emergency, cardiopulmonary resuscitation (CPR), opioid overdose, and administering naloxone. Comments suggested positive experiences with clinical simulation.

**Conclusion:**

Our study demonstrated that clinical simulation is feasible, acceptable, and effective for pre-health education. This study reiterates the value of hands-on, interprofessional, and collaborative learning in student engagement and learning outcomes.

## Introduction

Demographic trends indicate that future United States (U.S) workers will increasingly be persons of color: by 2050, one of every two U.S. workers will be African American, Hispanic, Asian American, Pacific Islander, or Native American. Yet physicians of color in the U.S. do not reflect this trend, comprising less than 20%: Black/African American 5.0%, Hispanic 5.8%, Native Americans less than 0.4%, and Pacific Islanders less than 0.2% [1].

This is troubling because health professionals from underrepresented groups are more likely to practice in underserved communities, thus increasing access to care in these communities [2]. Because many minority and rural neighborhoods have a shortage of physicians and less access to healthcare, increasing the supply of underrepresented physicians has been proposed as an intervention that may help to narrow the differences in health status [3]. Furthermore, with a projected shortage of 5 million college educated workers by 5 million by the year 2037, additional skills shortages of 362,000 nurses are projected [4].

Rural populations are also underserved and additionally face significant healthcare provider shortages that can impact healthcare outcomes [5,6]. West Virginia, for example, a largely rural state, ranks lowest in the country in healthcare outcomes, including having the highest mortality and the highest number of preventable deaths [6]. More broadly, there are 13.1 physicians per 10,000 residents in rural populations compared with 31.2 physicians per 10,000 in urban populations [7]. Additionally, rural populations may be considered more vulnerable. They are older, with individuals aged 65 and older comprising 18% of the rural population compared with 12% of the urban population. In addition, rural populations have higher poverty rates than urban populations and diabetes is 17% higher in rural populations than urban populations [8].

Therefore, efforts to increase the numbers of health professionals practicing in underserved areas can help to address disparities in outcomes of underserved patients. Such efforts require looking into the pipeline of those entering medical school in the United States to see what attracts them to a career in medicine. Many individuals pursuing a career in medicine share an interest in science. This is promising because as early as elementary school, American students start to express a strong interest in the sciences. However, this interest in science starts to wane as they progress through middle school and declines further in senior high school [9–11]. By the time students reach the undergraduate years, their interest in science has waned, frequently not taking on as much enriching science coursework and other experiences to prepare them for a career in medicine [12,13]. For those who do take science coursework, some feel discouraged by the lack of relevance to medicine and other health professions. Furthermore, lack of rural health professional role models limits exposure to health professions as viable career choices, further worsening the shortage [5].

In the United States, several enrichment programs have attempted to address this engagement in the sciences. For example, at Massachusetts General Hospital, a partnership was established between a cardiac anesthesia department and the urban senior high school student population to establish a Science, Technology, Engineering, Mathematics (STEM) enrichment program [14] with elements of research, mentorship, academic enhancement, motivation, and academic partnerships. However, such enrichment programs, as well as postgraduate training programs, tend to be clustered in urban areas, thus many may feel unprepared for the challenges in caring for underserved populations such as rural populations [5].

Learning is the process whereby knowledge is created through the transformation of experience [15]. Simulation is a form of experiential learning that takes place in a controlled environment with role-playing actors, manikins, and equipment used to replicate the clinical context of each case. Simulation creates a high-fidelity scenario with no risk of harm to real patients. Simulation has been effective in increasing both knowledge and skills among learners [16]. Several studies have compared the use of simulation to other learning methods. Most studies evaluated health professions students, providing evidence of improved understanding of pre-clinical basic science concepts, and the transition to clinical experiences [17–19]. Other studies have focused on interprofessional learning environments [20–22]. For example, simulation embedded into interprofessional training for stroke has led to increased understanding and interprofessional competencies [23,24]. Similarly, a pilot simulation study with pre-health students demonstrated the feasibility of experiential competency-based education by identifying greater resulting interprofessional competencies [25].

In addition, as a form of experiential learning, simulation can expose students to health professional roles and further help to rekindle interest or motivation to pursue the sciences as a pathway to health professional careers. Simulation, therefore, can translate academic knowledge into practical application, reducing the gap between classroom learning and real-world clinical performance [26]. Despite this potential, the research is still limited, particularly with regards to simulation in pre-health students. While several studies highlight the acceptability and learning improvements of simulations across medical and undergraduate students, none have focused on its effectiveness in pre-health students in rural health settings [27,28]

Our study aimed to determine the feasibility, acceptability, and effectiveness of a clinical simulation program as a learning strategy for pre-health students with various undergraduate majors.

## Methods

### Ethics

Our study underwent West Virginia University’s IRB review through the WV STEPS Simulation Center and was approved with study Protocol # 2308828386. Written informed consent was obtained from participants on the day of the simulation, prior to participation. Confidentiality was maintained, and data were de-identified upon data entry. The individuals who are identifiable in figures in this manuscript have given written informed consent (as outlined in the PLOS consent form) to publish these case details.

### Study setting

This study was conducted at the David and Jo Ann Shaw Center for Simulation Training and Education for Patient Safety (WV STEPS Simulation Center) within the Health Sciences Center of an academic university-affiliated medical center serving a largely rural patient population. The simulation center is designed to provide experiential learning opportunities for healthcare professionals, emphasizing inter-professional education among nurses, pharmacists, and physicians.

### Study design

This study uses a quasi-experimental pretest-posttest design, evaluating outcomes relevant to a one-day clinical simulation program. Simulations were carried out on eight weekend days, running from March 29, 2025, to May 4, 2025, and post-simulation data collection ended August 4, 2025. Each participant was involved in one of the weekend simulation days. The simulation environment was configured to replicate a diverse range of clinical scenarios, including one intraoperative case, two emergency critical care cases, and one neurological emergency case. These specific cases were chosen to represent conditions with a high incidence in the patient populations served by the medical center. Laerdel Learning Application (LLEAP) software was used to integrate with the Laerdal patient simulators.

### Participants

Participants were recruited for the simulation study through flyers distributed electronically across the institution with targeted outreach to pre-health student group leaders. Interested individuals indicated their availability through an online scheduling poll. Recruitment began on March 1, 2025 and ended May 4, 2025.

The inclusion criteria for participation were: currently enrolled at the institution; at least 18 years of age; and currently declared a pre-health area of concentration (though all undergraduate majors were welcome).

The exclusion criteria were: students already enrolled in a health professions program (e.g., medical or nursing students); students not currently enrolled at the institution; and under 18 years of age.

For this simulation study with one group of participants, a post-hoc analysis was conducted based on t tests, with a power = 80%, alpha = 0.05, and a Cohen’s d = 0.8. A minimum sample size of 28 participants was needed.

### Curriculum development

A one-day simulation curriculum was developed through a multi-step process to ensure a robust and relevant learning experience for pre-health students.

Before the research began, a needs assessment was conducted to identify key basic science concepts that were present in the clinical simulation scenarios and part of the undergraduate curriculum. This involved reviewing the existing undergraduate curriculum, particularly the pre-requisites for health professions admission, to pinpoint areas where students might benefit from enhanced understanding and practical application of topics such as genetics, biochemistry, physics, and physiology. This initial review was followed by a literature search to identify health issues affecting West Virginians and simulations that could be adapted for rural settings. Expert faculty were identified by Pre-health student organization leaders identified expert faculty to further inform on the most relevant clinical scenarios for pre-health students, focusing on high-acuity, high-risk situations commonly encountered in local populations.

For each of the four simulation scenarios (malignant hyperthermia, opioid overdose, stroke, and cardiac emergency), specific and measurable learning objectives that aligned with the identified basic science topics were created. These objectives addressed knowledge, skills, and attitudes related to managing these emergent clinical situations. A 20-item multiple-choice written knowledge test was developed to assess participants’ understanding of the core concepts related to the scenarios. Items covered the key learning objectives and included the recognition, initial management, and relevant pathophysiology of each clinical condition.

### Curriculum implementation

The developed curriculum was implemented as a single, full-day simulation program that included a lecture and simulation for a cohort of pre-health students.

The full-day event consisted of four distinct simulation scenarios (Table 1). Information about the data collection tools are found in supplemental materials (S1 Appendix). More details about the objectives and tasks are in the supplemental materials (S2 Appendix).

**Table 1:** Description and tasks for each of the four clinical scenarios.

Scenario	Description	Tasks
**Cardiac Arrest Scenario (CARD):**	This scenario provided training in the assessment and management of myocardial infarction and cardiac arrest, emphasizing high-quality CPR (Healthcare provider type) and team dynamics. Participants had opportunities to intubate in this emergency setting (Fig 1).	Attend to a patient with chest pain, consider EKG, lab values, imaging, and vital signs. Continue to consider the ABCs – airway, breathing and circulation, as changes happen throughout the case. Perform CPR as needed.
**Opioid Overdose Scenario (OP):**	This scenario focused on the recognition and initial management of opioid overdose, including the administration of naloxone and basic life support measures. Participants had the opportunity to intubate in this emergency setting.	Attend to a patient who is unresponsive, consider clues from the vital signs, the manikin’s appearance and responsiveness Administer naloxone intra-nasally Consider various modes of oxygen delivery for the patient, and how each method gradually increases oxygen delivery
**Intraoperative Scenario – Malignant Hyperthermia (MH):**	This scenario presented a critical event during a routine inguinal hernia repair in a pediatric patient, requiring participants to identify and manage an acute complication, malignant hyperthermia, within the operative environment. This scenario included performing tasks in the induction of anesthesia, a non-emergency intubation process, and monitoring of vital signs (Fig 2).	Administer oxygen, medications, perform endotracheal intubation. (Additional students can practice at extra intubation stations while the main space is prepared for “surgery”). Participants must monitor for vital sign changes (EtCO_2_) temperature) Learners must work as a team to stop the procedure, find, and administer dantrolene.
**Stroke Scenario (CVA):**	This scenario challenged participants to rapidly assess and manage a live, trained standardized patient presenting with stroke symptoms, including performing a neurological assessment and suggesting appropriate interventions. Participants were introduced to the National Institutes of Health Stroke Scale [29].	Learners must build rapport with the patient and family member (both live scenario actors) in order to get cooperation for information gathering and the physical exam. Learners must work together to obtain a physical exam and interpret imaging and lab results.

**Fig 1:**
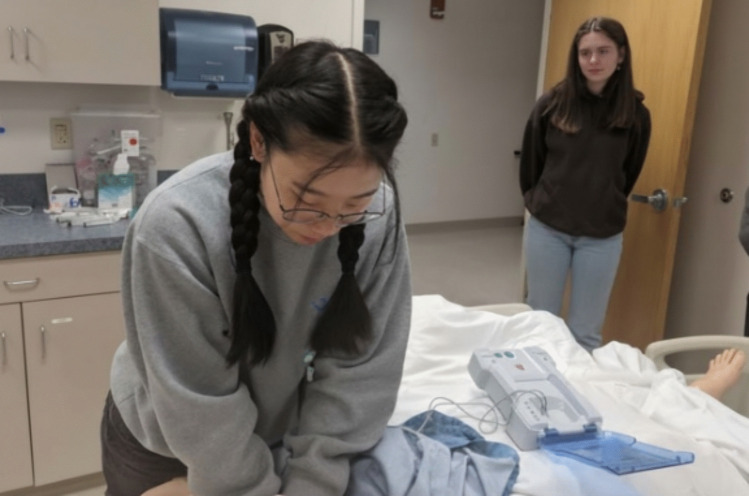
Participants take turns performing cardiopulmonary resuscitation on a manikin in the CARD simulation scenario (April 2025).

**Fig 2:**
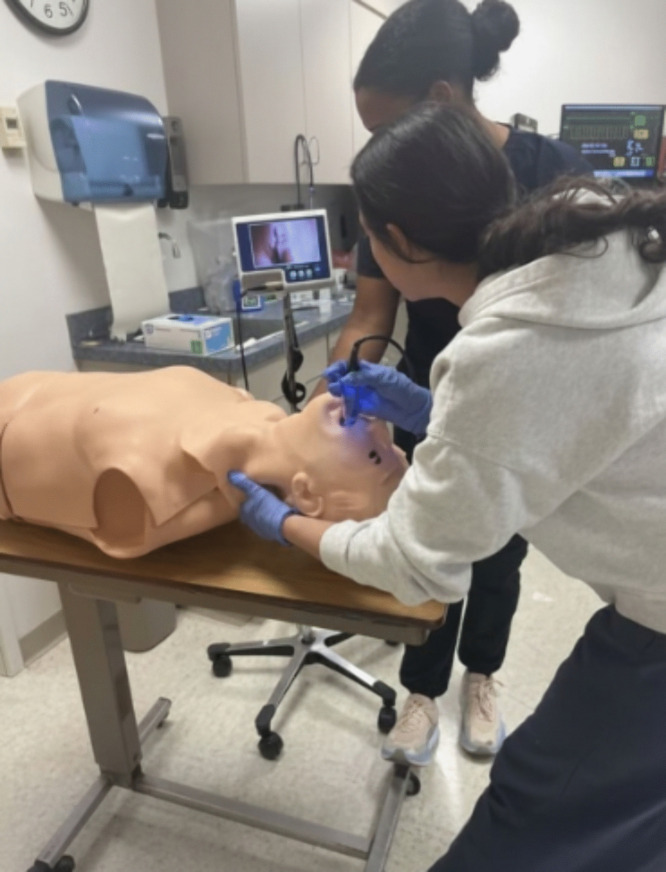
A simulation station with extra manikins for participants to demonstrate endotracheal intubation in the MH simulation scenario. Video-guided laryngoscopy was used to visualize the vocal cords (April 2025).

For each simulation scenario, participants worked in teams and were provided with a clinical context and patient information. Real-time feedback and guidance were provided by facilitators throughout the simulation, including discussion of interval radiographs, electrocardiograms, and laboratory information. The simulation environment included realistic manikins and equipment to enhance immersion and provide hands-on practice of critical skills, as well as interaction with a live standardized patient for the stroke simulation scenario. A structured debriefing session followed the simulations to reinforce learning points and address areas for improvement.

### Data collection methods

#### 1. Pre- and post-simulation assessments and surveys.

Knowledge assessments were administered to participants before, immediately after, and three months after the simulation to assess their understanding of key basic science concepts relevant to the simulated scenarios. These assessments evaluated factual recall, comprehension, and application of knowledge. The assessments were developed and validated based on established learning objectives for the curriculum, with 4–6 questions per scenario.

In addition to knowledge assessments, self-reported confidence in basic science and medical topics, attitudes about the simulation, and previous learning experiences were measured using five-point scaled items. These were administered before (pre-simulation survey) and after (post-simulation survey) the simulation (see Appendix for the survey questions).

#### 2. Qualitative data.

Following the simulations, students participated in group debriefing exercises. The PEARLS (Promoting Excellence And Reflective Learning in Simulation) framework was used to guide the debriefing process [30]. These prompts encouraged students to critically analyze their experiences, identify perceived challenges, and articulate key learning points. For instance, students were asked to describe moments of uncertainty, successful decision-making, or insights gained about their understanding of the principles involved. Due to the learner population (with varied health experiences), the intensity, and sensitivity of the scenarios, audiorecording was not conducted in order to encourage privacy and learner willingness to orally discuss their reactions to the scenario. Multiple simulation team members were present during debriefing for additional notetaking and observation. Post-simulation surveys included free-text comments about the simulation experience.

#### 3. Behavioral observation.

During the simulation, student decision-making processes were recorded to objectively assess their understanding and application of learned concepts within each scenario. This was done using a pre-defined checklist to document specific behaviors and actions taken by students during the simulation (See Appendix).

### Data analysis

Our first primary endpoint was knowledge assessment, comparing pretest, posttest, and 3-month posttest, analyzed descriptively and with paired t-tests. Similarly, our second primary endpoint, learner acceptability, using presurvey and postsurvey data, was analyzed with descriptive statistics and paired t tests. Our third primary endpoint was feasibility, measured participants who completed the simulation posttests and the 3-month posttests and analyzed with descriptive statistics. Qualitative data from debriefing and survey comments were analyzed with content analysis.

## Results

### Participant demographics

Forty-three pre-health students participated in the simulation and completed the pretest and immediate posttest. The average group size for each simulation day included 5 students. Thirty-six (36) students completed the 3-month posttest (83.7% response rate). The predominant majors were exercise physiology and biology. The majority were female and pre-medical students (Table 2). Additionally, the majority of students admitted to learning primarily with video and textbook (Table 3).

**Table 2:** Participant demographics.

Demographic variable	Number (%) of participants
Undergraduate Major	
Biology	9 (20.9%)
Exercise Physiology	9 (20.9%)
Neuroscience	4 (14%)
Biomedical Engineering	4 (9.3%)
Pre-Pharmacy	4 (9.3%)
Immunology & Microbiology	1 (2.3%)
Laboratory Diagnostics	2 (4.7%)
Forensics	3 (7.0%)
Public Health	1 (2.3%)
Electrical Engineering	1 (2.3%)
Biochemistry	4 (9.3%)
Health & Wellness	1 (2.3%)
Pre-Health Discipline	
Pre-Medicine	30 (69.8%)
Pre-Pharmacy	4 (9.3%)
Pre-Physician Assistant	9 (20.9%)
Sex	
Male	6 (14%)
Female	37 (86%)
Other	0 (0%)
Year in College	
Freshmen	14 (32.6%)
Sophomore	10 (23.3%)
Junior	10 (23.3%)
Senior	7 (16.3%)
Post-Bac	1 (2.3%)
Unknown	1 (2.3%)

**Table 3:** Pre-simulation survey questions of experiences with basic sciences.

Question	N	%
**What is the primary format in which you study basic sciences currently?		
**Textbook**	**20**	**46.5%**
**Videos**	**20**	**46.5%**
**Lectures**	**20**	**46.5%**
**Mobile app**	**5**	**11.6%**
**Talking to others who have already taken the class**	**1**	**2.3%**
**Flashcards**	**1**	**2.3%**
**Papers**	**1**	**2.3%**
**Questions**	**Mean**	**STE DEV**
**How important is it for you to learn basic sciences?	4.64	0.65
**How satisfied are you with this method currently (of studying basic sciences)?	4.05	0.58

### Simulation checklists (See Appendix)

Participants’ progression and actions through the simulation were monitored. All students completed 100% of the checklist items (See appendix)

All students (n = 43) completed the pretest and immediate posttest, demonstrating significant improvement in knowledge after the simulation (M1 = 10.6 + /-2.49; M2 = 16.4 + /-2.22; p < 0.001). Thirty-six students completed the 3-month posttest (drop-out rate of 16.3%), demonstrating long term retention in this latter group. (Table 4).

**Table 4:** Mean student scores on the pre- and post-simulation assessments. M1: pretest mean, M2: immediate posttest mean, M3: 3-month posttest mean.

	Mean Student Scores on the Pre- and Post-Simulation Assessments	
Assessment	Mean Score + /- STDEV	P value
Pre-simulation (n = 43)	M1 = 10.6 + /- 2.49	--
Post-simulation (short term retention: n = 43)	M2 = 16.4 + /- 2.22	M1 vs M2: P < 0.001
Post-simulation (long term retention: n = 36)	M3 = 16.2 + /- 2.57	M1 vs M3: P < 0.001

Table 5 provides a detailed, item-by-item descriptive analysis of the proportion of correct and incorrect responses across the pre-test and post-test. Questions that were the lowest on the pretest included question 1 [14%], about a genetic mutation and protein receptor, and some of the physics-based questions [11.6%]. Questions with the highest pre-test performance included neuroanatomy questions [97.7%]. For the immediate posttest, the biggest learning gains were in understanding level of responsiveness during anesthesia [48.3%], the receptor genetic mutation question [67.4], and the mathematics question [58.2%]. The smallest learning gains were in question 11 neuroanatomy physical exam question [−14%], and 19, a combined vital signs question [18.6%]. For the 3-month posttest, Questions 11 and 19 had higher learning gains [40.9%, 26.9%]. The greatest learning loss was question 15, about electrocardiogram (EKG) interpretation [−30.1%].

**Table 5:** Item by item descriptive analysis of pretest and posttest performance.

Q	Question Content	Correct Answer	Pretest % Correct (n = 43)	Immediate Posttest % Correct (n = 43)	PercentChange to correct from pretest	3-month Posttest % (n = 36)	PercentChange From immed.posttest
1	Receptor mutation (MH)	D	14	81.4	*+67.4*	65.7	*−15.7*
2	Pathophysiology Order of vital sign changes (MH)	B	58.2	67.4	*+9.2*	77.1	*+9.7*
3	Chemistry Cell Biology Physiology (MH)	A	42.3	95.3	*+53.0*	80	*−15.3*
4	Identify the cause/pathophysiology (MH)	C	72.1	100	*+27.9*	100	*0*
5	Cell biology Pathophysiology Pulmonary edema (OP)	B	60.5	86.4	*+25.9*	82.9	*−3.5*
6	Opioid antagonist pharmacology (OP)	A	72.1	95.3	*+23.2*	94.3	*−1*
7	Signs opioid overdose (OP)	D	97.7	100	*+2.3*	100	*0*
8	Neuroanatomy & function (CVA)	B	60.5	97.7	*+37.2*	91.4	*−6.3*
9	Pathophysiology (CVA)	D	83.7	93.0	*+9.3*	100	*+7*
10	Neuroanatomy (CVA)	D	97.7	97.7	*+0*	97.1	*−0.6*
11	Neuroanatomy (CVA)	A	30.2	16.2	*−14*	57.1	*+40.9*
12	Neuropharmacology (CVA)	C	74.4	97.7	*+23.3*	80	*−17.7*
13	Pathophysiology (CVA)	A	65.1	86.0	*+20.9*	91.4	*+5.4*
14	Pathophysiology Health Disparities (CARD)	B	23.2	58.1	*+34.9*	62.9	*+4.8*
15	Physiology (CARD)	D	16.2	93.0	*+76.8*	62.9	*−30.1*
16	Pathophysiology (CARD)	B	39.5	90.6	*+51.1*	82.9	*−7.7*
17	Physiology Anatomy (CARD)	A	86.4	97.7	*+11.3*	97.1	*−0.6*
18	Physics Physiology Mathematics (ALL)	D	11.6	69.8	*+58.2*	62.9	*−6.9*
19	Physics Engineering Physiology (OP)	B	11.6	30.2	*+18.6*	57.1	*+26.9*
20	Level of responsiveness (MH)	A	42.3	90.6	*+48.3*	80	*−10.6*

Abbreviations: MH: Malignant hyperthermia scenario. OP: opioid overdose scenario. CARD: Myocardial infarction and cardiac arrest scenario. CVA: stroke scenario.

### Surveys

Tables 6 and 7 represent data collected from surveys. Table 6 questions were administered once at the end of the simulation day. Table 6 compared pre-simulation confidence to post-simulation confidence ratings on certain knowledge areas and skills.

**Table 6:** Post-simulation survey course evaluation questions.– Responses were on a Likert scale of 1-5. (See appendix for answer choices).

Questions	Mean	STDEV
How clear were the course objectives?	4.36	0.62
How appropriate was the content to your level of learning?	4.39	0.76
How effective was this course in enhancing your knowledge of cellular processes?	4.06	0.956
How effective was this course in enhancing your knowledge of neuroanatomy?	4.02	0.801
How useful did you find the course overall?	4.77	0.480
How useful was the lecture component of the course?	4.58	0.663
How useful was the standardized patient component of the course?	4.77	0.427
How useful was the simulation component of the course?	4.84	0.374
How useful was this course in comparison to your prior educational experiences?	4.56	0.629

**Table 7:** Pre and Post Surveys comparison. Likert Scale 1-5: impact of clinical simulation on reported confidence. M1 represented average pre-simulation survey ratings, M2 represented average post-simulation survey ratings. Likert Scale: 1- Not confident, 2- Slightly confident, 3- Fairly confident, 4- Confident, 5- Very confident.

Question	p	M1 (STDEV)	M2 (STDEV)
How confident are you in your knowledge of cell biology?	0.167	3.16 (0.935)	3.44 (0.934)
How confident are you in your knowledge of physiology?	0.028*	2.62 (0.962)	3.07 (0.985)
How confident are you in your knowledge of chemistry?	0.529	3.24 (0.82)	3.34 (0.89)
How confident are you in your knowledge of physics?	0.771	2.26 (1.11)	2.37 (0.955)
How confident are you that you can apply your knowledge in a clinical setting?	0.0003*	2.62 (0.961)	4.0 (0.787)
How confident are you at identifying when someone is having a stroke?	< 0.0001*	2.21 (1.09)	3.98 (0.771)
How confident are you at identifying when someone is having a heart attack?	< 0.0001*	2.26 (1.01)	3.53 (1.03)
How confident are you at identifying if there is an intraoperative emergency?	< 0.0001*	1.47 (0.74)	4.11 (0.793)
How confident are you at identifying if someone needs cardiopulmonary resuscitation (CPR)?	<0.0001*	3.12 (1.19)	4.25 (0.79)
How confident are you at identifying if someone is having an opioid overdose?	< 0.0001*	2.31 (1.07)	4.47 (0.702)
How confident are you in using naloxone?	< 0.0001*	2.29 (1.34)	3.93 (0.910)
How confident are you in training others to use naloxone?	< 0.0001*	1.67 (1.1)	3.93 (1.07)

### Course usefulness

Content analysis of the three open ended questions on course usefulness revealed several themes from participants’ responses: 1) Participants liked the practicality of simulation, 2) participants enjoyed the intubations, 3) participants liked having copies of the lecture slides for reference, 4) participants disliked the waiting time between the second and third simulations, and 5) participants brought varied backgrounds and experiences to simulation day.

#### Simulation was a practical application of what they just learned.

Participants stated that they preferred the “hands on learning” and the opportunity to “apply what was learned in lecture.” One participant stated that the simulation “was very useful and really helped to understand concepts.” More than half of participants identified the cardiac and opioid overdose as their favorite simulations, finding them the most “useful.” Another participant liked “getting to see” what they “were taught firsthand.” Several participants indicated that they wanted “more types of scenarios” and to “include more simulation.”

#### Intubations.

Several participants liked “learning to intubate” and desired “more opportunities to intubate.” One participant did not like learning “to use the camera” (video laryngoscope) for the initial group demonstration nor to guide intubation and would prefer to “instead” use “just direct,” or without video guidance. Suggestions for the future included adding “as many clinical applications like the intubation as possible. This was the most important and cool thing.”

#### Having a copy of the lecture slides.

Participants appreciated having a hard “copy of the lecture slides” during the lecture and simulation portions, and even after simulation day.

#### Waiting time between the first two simulations and the last two simulations.

Participants preferred time spent in simulations to other learning modalities. Participants from simulation days with higher attendance did not like having unstructured free time between the second and third simulation. Participants suggested having “more than one instructor for bigger groups so they can work simultaneously”, saying this could result in “waiting less” and “slightly better flow” between simulations and would create “smaller groups.”

#### This was a new and unusual learning experience for many pre-health learners, although some likely had prior experience with simulation content.

Most participants indicated that they had never “been in a clinical setting before this course.” Some participants thought “some of the questions on the exams were more detailed than the lecture material” or that during the actual simulation some wanted to “go a little more in-depth on the simulations such as discussing why things are occurring,” long before the debriefing session. One participant would have liked “a schedule so we can come more prepared.” Another participant indicated that “The least useful aspect for me was probably learning about how malignant hyperthermia occurs because I will not encounter that in my future career.”

On the other hand, some participants likely had prior healthcare experience and wanted “more simulations where the participants work alone to try to assess their skills without the instructors.” Because a few participants had already learned CPR and opioid overdose for their healthcare jobs, they would have liked an additional “info email” prior to simulation day about the simulation content details “in case someone has learned everything” already.

### Group debriefing session

From the content analysis of participant debriefing comments, six themes emerged: 1) Learning about the vital signs, 2) reacting to new and useful scenario situations, 3) addressing complexities with chest pain, 4) enjoying intubations, 5) intense focus on medication details, and 6) unique features of the neurological simulation.

#### Vital signs.

Participants indicated that they learned a lot about vital signs in each case, allowing them to consider “normal vital signs,” “the ABCs” (airway, breathing and circulation) and “how to read the monitor.” Several participants agreed that one approach to intraoperative emergencies can be described as “correlation between vital signs and things.” In addition to “high temperature,” “high CO_2_ indicated malignant hyperthermia” according to multiple participants, with agreement from other participants. Lack of a pulse suggested the patient “needed CPR.” For the opioid overdose scenario, one participant indicated that “there was a gas exchange issue, which meant fluid in the lungs, because respiratory rate was not high enough even with 100% oxygen.”

#### New situation for them, yet applicable.

A common participant reaction was that “A lot of us didn’t know what we were doing at first.” Some thought the simulation “felt harder than I thought it would be.” Participants realized that they had to be “prepared for anything,” because patients could be “responsive at first” before suddenly becoming unresponsive. One approach that participants agreed on was “more or less trying to work backwards,” that is, retracing the steps and tasks in reverse order to see what happened. Despite an initial overwhelming introduction to the simulations, participants indicated that they thought the cases were useful. For example, participants agreed that the opioid overdose case was “very applicable to the setting,” and “even outside of the clinical” setting.

#### What type of chest pain.

Participants agreed that chest pain can present in different ways. For example, participants agreed to “check to make sure not GERD” and “not dismissing” the patient’s concerns. Participants’ learning points included “taking what” patients “say seriously;” or else, for example, “women can get dismissed or delayed” in care.

#### Intubations.

Participants liked that they “learned intubations all day”. Learning points included “how to intubate” and recognizing when situations called for “intubations if needed.”

#### Medications.

While the scenario objectives did not require memorizing specific medications, participants were interested and focused on whether they knew these names. Sometimes participants would discuss events from the scenario but mentioned that they “forgot drug names.” Some participants thought the simulation was “hard because they forgot drug names” or they were “trying to figure out what medications” to use.

#### Unique features of neurological simulation.

The neurological simulation differed from the others in that a live standardized patient was used, instead of a manikin. Participants liked to use “the NIH Stroke Scale.” [29] This simulation was important in learning how to do the tests, “because reading the scale was not even enough.” In addition, this simulation allowed participants to consider “how to communicate with a live patient.” They “liked doing the exam and getting to ask questions” in communicating with the patient and family. Participants also agreed that an “intense discussion would be” addressed to “improve diet and exercise and give an example” of what that “would be in the patient’s life.”

## Discussion

Clinical simulation was a feasible, acceptable, and effective learning tool for pre-health students. Results from the knowledge and skill assessments suggest that this clinical simulation curriculum was effective in enhancing knowledge of key scientific concepts and learners’ ability to perform clinical skills. Learner ratings and comments suggest that the curriculum was acceptable and that more simulation opportunities, especially with intubation, were desired. They found the sessions engaging, relevant to their future careers, and expressed a desire for more such opportunities. Primarily, participants demonstrated significant improvement in the knowledge and skill assessments, suggesting that the program was effective. This aligns with several studies that have compared using simulation with other learning methods. Health professions students frequently show evidence of improved understanding of pre-clinical basic science concepts and transitioning to clinical experiences [19–21]. Another study demonstrated that group activity in an introductory biology course improved learning gains [31].

Yet the test accuracy varied across questions and time points. For instance, while overall performance would suggest that participants had prior exposure to neuroscience, the neurological physical exam question about one sided stroke was missed in the pretest and immediate post-test but improved in long term follow up. The question about the level of responsiveness with the level of sedation was commonly answered incorrectly on pretests and improved on the posttests, suggesting that the general public may not understand the different levels of sedation and anesthesia, but with education, understanding can improve. Commonly missed immediate and long-term posttest questions include those requiring physiological understanding of multiple vital signs, such as regarding what affects pulse oximetry and initial signs of malignant hyperthermia. The most common question answered correctly on immediate posttest but not on the long-term post-test was the question about the genetic mutation responsible for malignant hyperthermia. These results align with those of a previous study with pre-health students at a community college, which suggested that role playing the components of protein synthesis was effective in immediate learning gains on a 5-item knowledge test, but those gains were not as significant at long-term follow up [32]. The study also shows that certain questions, such as those related to protein mutations, and cellular processes were less likely to be retained in long-term follow up. Taken together, these findings suggest that some concepts may benefit from long term study, and that a single simulation day may not be enough to address certain concepts.

There are some studies that demonstrate feasibility, acceptance, or effectiveness of various learning methods in pre-health students. A simulation study with GEMMS-PA program students at the University at Salford for pre-health students interested in medicine and physician assistant careers demonstrated acceptability with positive feedback supporting career development and preference for simulation over lecture formats for learning [27]. Another study about undergraduate students and simulation effectively improved understanding of physiological concepts and demonstrated the acceptability of simulation-based case studies over paper-based case studies [28]. These findings are similar to this study as this study demonstrated significant self-reported and assessment-based learning gains in physiological understanding. Overall, these findings suggest that simulation-based education holds promise as a valuable pedagogical approach for students pursuing future healthcare careers.

This study is unique in that pre-health students at all college levels were included, with various undergraduate majors, and in a center with opportunities for rural health exposure. This study not only demonstrated short term feasibility, acceptability, and effectiveness, but also demonstrated long-term effectiveness. In addition, participants have found simulation to be useful, especially the opioid overdose simulation, which could have implications for undergraduate students’ campus access to naloxone to help with emergencies [33]. Finally, this study considered evaluating the thought processes of participants as they experienced each simulation, noting reflections and feedback qualitatively.

This simulation is an example of an enrichment program that was feasible, enjoyable, and effective, even as a one-day event. Thus, undergraduate curricula can incorporate similar experiential opportunities to further demonstrate the relevance of basic science topics to healthcare. Similar programs can help address healthcare provider and skill shortages by providing experiential learning and exposure to practical clinical skills, such as opioid overdose training. This could then benefit the healthcare workforce, particularly in underserved populations [34,35]. Other efforts to address the healthcare workforce shortages include looking to international medical graduates (IMGs), who currently comprise 25% of the physician workforce in the United States [36]. Increasing health professions training programs in underserved communities can be another strategy. However, the strategy that this simulation program directly addresses is the pipeline of pre-health students. Our study was an experiential opportunity for pre health students to demonstrate increased confidence, realize what they are getting into, and enhance career goal setting, so that they can make informed decisions about entering the health professions [28,37].

### Limitations

Despite the promising findings, this study has some limitations. Demographic information regarding previous healthcare was not collected, despite qualitative data indicating that students had varying backgrounds in healthcare. This simulation could be particularly impactful for students with less prior exposure to healthcare settings, although this was difficult to account for without this demographic data Also, it is unknown whether participants had post-simulation exposures related to the assessment topics. If they did, this could have impacted their performance on the 3-month post assessment.

All participants were from a single institution, limiting the generalizability of the results. Additionally, reliance on self-reported confidence and satisfaction scores could be subject to bias. Future research with more objective measures, and the use of comparison groups will further strengthen these findings.

Another limitation is the 16.3% 3-month posttest non-response rate. While this non-response rate is moderate, the possibility of non-response bias must be considered. Non-response bias is a potential limitation when a portion of the sample does not provide data, as the responding group may not accurately represent the intended population. To investigate this possibility, we conducted a non-response bias analysis. Responders were compared to non-responders on baseline characteristics and pre-simulation scores that were available for the entire cohort. Independent samples t-tests and chi-square tests revealed no statistically significant differences across these variables (p > 0.05). While this comparison suggests that non-response bias is not a major threat to the validity of the findings, it is impossible to account for all potential unmeasured differences. It could be that some students had graduated and changed university accounts, or that many of the non-responding students did not respond during the summer months. The results should be interpreted with this limitation in mind.

Furthermore, this simulation program was conducted with all participants receiving the same educational exposure and analyzed as one group. Demonstrated learning gains post-simulation could be due to the experience from taking the assessments. Therefore, further studies with at least two groups with different educational content or strategies could better characterize the effectiveness of the simulation in one group compared to the other group.

Finally, several statistical tests were implemented, thus increasing the chance of a Type 1 error. With a Bonferroni correction, the significance level changed to p < 0.025. Many of our findings still are found to be significant, but these findings should be interpreted with caution.

### Conclusion

This study demonstrated that clinical simulation was feasible, acceptable, and effective for pre-health student education. By providing a safe and engaging environment for hands-on learning, clinical simulation can enhance knowledge, skills, and confidence, better preparing future healthcare professionals for the demands of a career in healthcare. Continued research and development in this area are crucial to optimize simulation-based education integration into pre-health education and maximize its positive impact on the quality of healthcare delivery. This study reiterates the importance of hands-on, interprofessional, and collaborative learning in enhancing student engagement and learning outcomes, and increasing the pipeline of students going into the health professions.

## Supporting information

S1 Appendix Data collection tools. Data collection instruments utilized in the study. Includes the baseline survey questionnaire, the multiple-choice knowledge assessment administered for both pretest and posttest, and observational checklists used during evaluation phases.
(DOCX)

S2 Appendix Simulation guides. Standardized simulation scenario guides and templates. Includes four distinct clinical simulation templates with each scenario detailing setup instructions, learning objectives, critical action checklists, and debriefing frameworks.
(DOCX)

## References

[1] Association of American Medical Colleges. Diversity in Medicine: Facts and Figures 2019. [cited 2025 Sept 20]. Available from: https://www.aamc.org/data-reports/workforce/report/diversity-medicine-facts-and-figures-2019

[2] Bruza-Augatis M, Coplan B, Sethi W, McDaniel MJ. The influence of patient care, shadowing, and volunteer experience on diverse applicant matriculation into physician assistant/associate programs. J Physician Assist Educ. 2024;35(2):136–43. doi: 10.1097/JPA.0000000000000563 37962338

[3] Chan KS, Parikh MA, Thorpe RJ Jr, Gaskin DJ. Health care disparities in race-ethnic minority communities and populations: does the availability of health care providers play a role? J Racial Ethn Health Disparities. 2020;7(3):539–49. doi: 10.1007/s40615-019-00682-w 31845286 PMC7231628

[4] Smith N, Van Der Werf M, Adelson M, Strohl J. Falling behind: how skills shortages threaten future jobs. Georgetown University’s Center on Education and the Workforce; 2025.

[5] Nielsen M, D’Agostino D, Gregory P. Addressing rural health challenges head on. Mo Med. 2017;114(5):363–6. 30228634 PMC6140198

[6] Geek M. Best and Worst States for Health Care in the US in 2025. [cited 2025 Aug 17]. Available from: Moneygeek.com/resources/top-states-healthcare

[7] About Rural Health Care. National Rural Health Association; 2025 [cited 2025 Sept 20]. Available from: https://www.ruralhealth.us/about-us/about-rural-health-care#:~:text=The%20obstacles%20faced%20by%20health,live%20in%20poverty.%5B5%5D

[8] Coughlin SS, Clary C, Johnson JA, Berman A, Heboyan V, Benevides T, et al. Continuing challenges in rural health in the United States. J Environ Health Sci. 2019;5(2):90–2. 32104722 PMC7043306

[9] Sorge C. What happens? Relationship of age and gender with science attitudes from elementary to middle school. Sci Educ. 2007;16(2):33–7.

[10] Potvin P, Hasni A. Analysis of the decline in interest towards school science and technology from grades 5 through 11. J Sci Educ Technol. 2014;23(6):784–802. doi: 10.1007/s10956-014-9512-x

[11] Zhang J, Li M. Motivation, NAEP performance, and choice of a STEM major: the role of domain-specific academic. Identity, self-efficacy and interest: a synthesis report; 2023 [cited 2025 Sept 20]. Available from: https://files.eric.ed.gov/fulltext/ED655799.pdf

[12] Prescod DJ, Daire AP, Young C, Dagley M, Georgiopoulos M. Exploring negative career thoughts between STEM‐declared and STEM‐interested students. J Employ Couns. 2018;55:166–75. doi: 10.1002/joec.12096

[13] An analysis of the medical school pipeline: a high school aspirant to applicant and enrollment view. AAMC Anal Brief. 2014;14(3).

[14] Woodward E, Lai Y, Egun C, Fitzsimons MG. How cardiac anesthesiology can help “STEM” the tide of under-representation of minorities in science and medicine. J Cardiothorac Vasc Anesth. 2018;32(2):631–5. doi: 10.1053/j.jvca.2017.06.031 29366746

[15] Kolb DA. Experiential learning experience as the source of learning and development. Englewood Cliffs (NJ): Prentice Hall; 1984.

[16] Chima AM, Koka R, Lee B, Tran T, Ogbuagu OU, Nelson-Williams H, et al. Medical simulation as a vital adjunct to identifying clinical life-threatening gaps in austere environments. J Natl Med Assoc. 2018;110(2):117–23. doi: 10.1016/j.jnma.2017.12.003 29580444

[17] Zheng J, Lapu R, Khalid H. Integrating high-fidelity simulation into a medical cardiovascular physiology curriculum. Adv Med Educ Pract. 2020;11:41–50. doi: 10.2147/AMEP.S230084 32021542 PMC6970253

[18] Alluri RK, Tsing P, Lee E, Napolitano J. A randomized controlled trial of high-fidelity simulation versus lecture-based education in preclinical medical students. Med Teach. 2016;38(4):404–9. doi: 10.3109/0142159X.2015.1031734 25897707

[19] Jowsey T, Petersen L, Mysko C, Cooper-Ioelu P, Herbst P, Webster CS, et al. Performativity, identity formation and professionalism: ethnographic research to explore student experiences of clinical simulation training. PLoS One. 2020;15(7):e0236085. doi: 10.1371/journal.pone.0236085 32730277 PMC7392231

[20] Lockeman KS, Lanning SK, Dow AW, Zorek JA, DiazGranados D, Ivey CK, et al. Outcomes of introducing early learners to interprofessional competencies in a classroom setting. Teach Learn Med. 2017;29(4):433–43. doi: 10.1080/10401334.2017.1296361 28281832

[21] Moro C, Phelps C. Encouraging study in health sciences: informing school students through interprofessional healthcare simulations. Simul Healthc. 2024;19(3):144–50. doi: 10.1097/SIH.0000000000000732 37255339

[22] Wilson M, Bray BS, Remsberg CM, Kobayashi R, Richardson B. Interprofessional education on opioid use and pain identifies team-based learning needs. Curr Pharm Teach Learn. 2021;13(4):429–37. doi: 10.1016/j.cptl.2020.11.011 33715807

[23] MacKenzie D, Creaser G, Sponagle K, Gubitz G, MacDougall P, Blacquiere D, et al. Best practice interprofessional stroke care collaboration and simulation: the student perspective. J Interprof Care. 2017;31(6):793–6. doi: 10.1080/13561820.2017.1356272 28862889

[24] Pinto C, Possanza A, Karpa K. Examining student perceptions of an inter-institutional interprofessional stroke simulation activity. J Interprof Care. 2018;32(3):391–4. doi: 10.1080/13561820.2017.1405921 29265894

[25] Porter KJ, Nandan M, Varagona L, Maguire MB. Effect of experiential competency-based interprofessional education on pre-professional undergraduate students: a pilot study. J Allied Health. 2020;49(2):79–85. 32469366

[26] Powers K, Chen H, NSF. Exploring how engineering internships and undergraduate research experiences inform and influence college students’ career decisions and future plans. National Science Foundation; 2025. Available from: https://par.nsf.gov/servlets/purl/10076373#:~:text=The%20effectiveness%20of%20these%20high,decisions%20regarding%20post%2Dgraduation%20careers

[27] Amar K, Jones M, Connell N, Mayo D, Forde L, Sayan BS, et al. Enhancing career development for biomedical sciences students: leveraging simulations to support patient-facing careers. Curr Res Physiol. 2025;8:100150. doi: 10.1016/j.crphys.2025.100150 40584696 PMC12205311

[28] Chen H, Kelly M, Hayes C, van Reyk D, Herok G. The use of simulation as a novel experiential learning module in undergraduate science pathophysiology education. Adv Physiol Educ. 2016;40(3):335–41. doi: 10.1152/advan.00188.2015 27445282

[29] NINDS. NIH Stroke Scale. National Institute of Neurological Disorders and Stroke; 2025 [cited 2025 Sept 20]. Available from: https://www.ninds.nih.gov/health-information/stroke/assess-and-treat/nih-stroke-scale#:~:text=Developed%20through%20research%20supported%20by%20NINDS%2C%20the,and%20perform%20several%20physical%20and%20mental%20tests

[30] Eppich W, Cheng A. Promoting Excellence and Reflective Learning in Simulation (PEARLS): development and rationale for a blended approach to health care simulation debriefing. Simul Healthc. 2015;10(2):106–15. doi: 10.1097/SIH.0000000000000072 25710312

[31] Marbach-Ad G, Rietschel CH, Saluja N, Carleton KL, Haag ES. The use of group activities in introductory biology supports learning gains and uniquely benefits high-achieving students. J Microbiol Biol Educ. 2016;17(3):360–9. doi: 10.1128/jmbe.v17i3.1071 28101262 PMC5134939

[32] Avadhani M. Role play as an effective engagement technique in an introductory biology class. TALES. 2024;4(1). doi: 10.52938/tales.v4i1.3211

[33] Freibott CE, McCann NC, Biondi BE, Lipson SK. Interventions to increase naloxone access for undergraduate students: a systematic review of the literature. J Am Coll Health. 2024;73(6):2398–406. doi: 10.1080/07448481.2023.2299404 38227912 PMC11250916

[34] O’Donnell J, Tanz LJ, Gladden RM, Davis NL, Bitting J. Trends in and characteristics of drug overdose deaths involving illicitly manufactured fentanyls — United States, 2019–2020. MMWR Morb Mortal Wkly Rep. 2021;70:1740–6. 10.15585/mmwr.mm7050e3 34914673 PMC8675656

[35] He Z, Heess JM, Young T, Lei Z. Lessons for future pandemics: temporal evolution and rural-urban variations in the impacts of the COVID-19 on opioid use treatment. PLoS One. 2024;19(9):e0310386. doi: 10.1371/journal.pone.0310386 39269961 PMC11398672

[36] American Medical Association. About the International Medical Graduates Section (IMGS); 2025 [cited 2025 Sept 20]. Available from: https://www.ama-assn.org/member-groups-sections/international-medical-graduates-section-imgs/about-international-medical

[37] Strekalova YA, Liu-Galvin R, Border S. Summer research internship curriculum to promote self-efficacy, researcher identity, and peer-to-peer learning: retrospective cohort study. JMIR Form Res. 2025;9:e54167. doi: 10.2196/54167 PMC11809269 39899006

