# Supplementary material for: Advancing health equity through simulation-based education for pre-health students
Source: PLoS One. 2026 Aug 12;21(8):e0354377. doi: 10.1371/journal.pone.0354377 (PMC13465807; doi:10.1371/journal.pone.0354377)
Supplement: Supplementary file 1 [file pone.0354377.s001.docx]

**PRE-SURVEY**

**Year in college	Unique ID _____________________**

**(General attitudes toward basic science education)**

1. **How important is it to you to learn basic sciences (biological and physical sciences) outside of the lecture or lab?**

1- Not important	2- Slightly important	3- Fairly important	4- Important	5- Very important

2. **What is the primary format in which you study basic sciences currently (textbook, mobile app, videos, etc.)?**
3. **How satisfied are you with this method?**

1- Very dissatisfied	2- Dissatisfied	3- Neither	4- Satisfied	5- Very satisfied

4. **How confident are you in your knowledge of cell biology?**

1- Not confident	2- Slightly confident	3- Fairly confident 4- Confident 5- Very confident

5. **How confident are you in your knowledge of physiology?**

1- Not confident 2- Slightly confident	3- Fairly confident	4- Confident	5- Very confident

6. **How confident are you in your knowledge of chemistry?**

1- Not confident 2- Slightly confident	3- Fairly confident	4- Confident	5- Very confident

7. **How confident are you in your knowledge of physics?**

1- Not confident 2- Slightly confident	3- Fairly confident 4- Confident	5- Very confident

8. **How confident are you that you can apply your knowledge in a clinical setting?**

1- Not confident 2- Slightly confident	3- Fairly confident	4- Confident	5- Very confident

9. **How confident are you at identifying when someone is having a stroke?**

1- Not confident 2- Slightly confident	3- Fairly confident	4- Confident	5- Very confident

10. **How confident are you at identifying when someone is having a heart attack?**

1- Not confident 2- Slightly confident	3- Fairly confident	4- Confident	5- Very confident

11. **How confident are you at identifying if there is an intraoperative emergency?**

1- Not confident 2- Slightly confident	3- Fairly confident	4- Confident	5- Very confident

12. **How confident are you at identifying if someone needs cardiopulmonary resuscitation (CPR)?**

1- Not confident 2- Slightly confident	3- Fairly confident 4- Confident	5- Very confident

13. **How confident are you at identifying if someone is having an opioid overdose?**

1- Not confident 2- Slightly confident	3- Fairly confident	4- Confident	5- Very confident

14. **How confident are you in using naloxone?**

1- Not confident 2- Slightly confident	3- Fairly confident	4- Confident	5- Very confident

15. **How confident are you in training others to use naloxone?**

1- Not confident 2- Slightly confident	3- Fairly confident	4- Confident	5- Very confident

**POST SURVEY (to be completed at end of simulation day)**

**Year in college	Unique ID _____________________**

1. **How confident are you in your knowledge of cell biology?**

1- Not confident 2- Slightly confident	3- Fairly confident	4- Confident	5- Very confident

2. **How confident are you in your knowledge of physiology?**

1- Not confident 2- Slightly confident	3- Fairly confident 4- Confident 5- Very confident

3. **How confident are you in your knowledge of chemistry?**

1- Not confident 2- Slightly confident	3- Fairly confident	4- Confident	5- Very confident

4. **How confident are you in your knowledge of physics?**

1- Not confident 2- Slightly confident	3- Fairly confident	4- Confident	5- Very confident

5. **How confident are you that you can apply your knowledge in a clinical setting?**

1- Not confident 2- Slightly confident	3- Fairly confident	4- Confident	5- Very confident

6. **How confident are you at identifying when someone is having a stroke?**

1- Not confident 2- Slightly confident	3- Fairly confident	4- Confident	5- Very confident

7. **How confident are you at identifying when someone is having a heart attack?**

1- Not confident 2- Slightly confident	3- Fairly confident	4- Confident	5- Very confident

8. **How confident are you at identifying if there is an intraoperative emergency?**

1- Not confident 2- Slightly confident	3- Fairly confident	4- Confident	5- Very confident

9. **How confident are you at identifying if someone needs cardiopulmonary resuscitation (CPR)?**

1- Not confident 2- Slightly confident	3- Fairly confident	4- Confident	5- Very confident

10. **How confident are you at identifying if someone is having an opioid overdose?**

1- Not confident 2- Slightly confident	3- Fairly confident	4- Confident	5- Very confident

11. **How confident are you in using naloxone?**

1- Not confident 2- Slightly confident	3- Fairly confident	4- Confident	5- Very confident

12. **How confident are you in training others to use naloxone?**

1- Not confident 2- Slightly confident	3- Fairly confident	4- Confident	5- Very confident

**OVERALL EVALUATION AT END OF SIMULATION DAY**

13. **How clear were the course objectives?**

1- Not clear	2- Slightly clear	3- Fairly clear	4- Clear	5- Very clear

14. **How appropriate was the content to your level of learning?**

1- Not appropriate	2- Slightly appropriate	3- Fairly appropriate	4- Appropriate	5- Very appropriate

15. **How effective was this course in enhancing your knowledge of cellular processes?**

1- Not effective	2- Slightly effective	3- Fairly effective	4- Effective	5- Very effective

16. **How effective was this course in enhancing your knowledge of neuroanatomy?**

1- Not effective	2- Slightly effective	3- Fairly effective	4- Effective	5- Very effective

17. **How useful did you find the course overall?**

1- Not useful	2- Slightly useful	3- Fairly useful	4- Useful	5- Very useful

18. **How useful was the lecture component of the course?**

1- Not useful	2- Slightly useful	3- Fairly useful	4- Useful	5- Very useful

19. **How useful was the standardized patient component of the course?**

1- Not useful	2- Slightly useful	3- Fairly useful	4- Useful	5- Very useful

20. **How useful was the simulation component of the course?**

1- Not useful	2- Slightly useful	3- Fairly useful	4- Useful	5- Very useful

21. **How useful was this course in comparison to your prior educational experiences?**

1- Not useful	2- Slightly useful	3- Fairly useful	4- Useful	5- Very useful

22. **What did you find the most useful about this course?**
23. **What did you find to be the least useful aspect of this course?**
24. **How can we make the course more useful? Please be as specific as possible.**
25. **Is there another topic that you would like to see a course specifically developed around? Please elaborate.**

Appendix

**Written Test**

1. The most common protein mutation in cells that results in malignant hyperthermia (MH) is in the:
   A. Sodium-potassium ATPase
   B. Sodium-Glucose transporter
   C. Acetylcholine receptor
   D. Ryanodine receptor
2. The earliest sign(s) of malignant hyperthermia (MH) is (are):
   A. Tachycardia (increased heart rate)
   B. Rising end-tidal CO2 (EtCO2)
   C. Elevated Temperature
   D. Rhabdomyolysis (muscle tissue breakdown)
   E. Both A & B
3. The element that is responsible for the body's deregulation in a malignant hyperthermia (MH) crisis is _____________.
   A. calcium
   B. magnesium
   C. sodium
   D. phosphorus

4. What is the first step the team should take in caring for a patient in a malignant hyperthermia (MH) crisis?

A. Call for a stat chest x-ray.

B. Apply heating pads to patient.

C. Stop all anesthetic agents and administer 100% oxygen.

D. Continue with the surgery.

### 5. During your pre-op assessment, you find your patient has pulmonary edema (excess fluid build-up in the lungs). How will this affect the diffusion of your gases?

### A. Increase distance across membrane, and increase diffusion

### B. Increase distance across membrane and decrease diffusion

### C. Decrease distance across membrane and increase diffusion

### D. Decrease distance across membrane and decrease diffusion

6. What is naloxone used for?

A. To reverse the effects of an opioid overdose (heroin, fentanyl, methadone, etc.)

B. To reverse the effects of a methamphetamine overdose

C. To reverse the effects of a cocaine overdose

D. To reverse the effects of any overdose

E. Don’t know/not sure

7. All of the following are signs of opioid overdose EXCEPT:

A. Unconsciousness

B. Unresponsiveness

C. Respiratory depression or arrest (not breathing)

D. Impulsive

8. A patient's MRI imaging shows damage to the cerebellum a week after the patient suffered a stroke. What assessment findings would correlate with this MRI finding?

A. Vision problems

B. Balance impairment

C. Language difficulty

D. Impaired short-term memory

9. What are the two very general types of stroke?

A. Venous (veins) and myocardial (heart muscle)
B. Hemorrhagic (bleeding) and venous (veins)
C. Ischemic (lack of blood flow) and myocardial (heart muscle)
D. Hemorrhagic (bleeding) and ischemic (lack of blood flow)

10. A patient is demonstrating signs and symptoms of stroke. The patient reports loss of vision. What area of the brain do you suspect is affected based on this finding?

A. Brain stem

B. Hippocampus

C. Parietal lobe

D. Occipital lobe

11. A patient has right sided brain damage from a stroke. Which of the following would not be a sign of this type of stroke?

A. Right side hemiplegia (weakness and paralysis)

B. Confusion on date, time, and place

C. Unilateral neglect (inattentive to stimuli on one side of body or environment)

D. Right-sided imbalance

12. In order for tissue plasminogen activator (tPA) to be most effective in the treatment of stroke, it must be administered?

A. 6 hours within the time frame of stroke symptoms

B. 3 hours before the time frame of stroke symptoms

C. 3 hours within the time frame of stroke symptoms

D. 12 hours before the time frame of stroke symptoms

13. What is happening when the inside of a narrowing blood vessel is accumulating plaque causing a temporary spasm (narrowing of the blood vessels causes by persistent contraction of the vessels)?

A. Transient ischemic attack (TIA)

B. Stroke

C. Subdural hematoma (swelling of blood)

14. The nurse is caring for a female patient who is having an acute heart attack (myocardial infarction – MI). Which of the following symptoms is more common in females who are experiencing a heart attack (myocardial infarction – MI), leading to delayed diagnosis?

A. Bradycardia (slow heart rate)

B. Heartburn

C. Cough

D. Chest pain

15. A patient with suspected heart attack (myocardial infarction – MI) is triaged in the emergency department. Upon review of the EKG, the nurse would expect which of the following findings consistent with the suspected condition?

A. Increased PR interval

B. Widened QRS Complex

C. Increased amplitude of P wave

D. Increased ST segment amplitude

16. You ordered lab tests on a patient presenting with complaints of chest pain for 6 hours.  Which of the following abnormal values would you expect to see if the patient’s chest pain is cardiac (heart)-related?

A. Low potassium of 3.1

B. Elevated troponin of 4.5

C. Low hemoglobin of 7.3

D. Low glucose of 65

17. Why is allowing complete chest recoil important when performing high-quality CPR?

A. The heart will adequately refill between compressions.

B. There will be a reduction of rescuer fatigue.

C. The rate of chest compressions will increase.

D. It will reduce the risk of rib fractures.

18. If the diameter of any intravenous (IV) catheter is doubled, flow through the catheter will

A. Decrease by factor of 2
B. Decrease by factor of 4
C. Increase by factor of 8
D. Increase by factor of 16
E. Increase by factor of 32

19. Each of the following factors may lead to erroneous readings using pulse oximetry EXCEPT

A. electrocautery (electric-powered tool used on tissue during surgery)

B. high cardiac output states

C. infrared lights near the sensor

D. intravenous (IV) dyes

E. severe hemodilution (decreased concentration of blood components)

20. Under which of these states is the patient least responsive?

A. General anesthesia
B. Minimal sedation
C. Moderate sedation
D. Deep sedation

**Simulation Checklists**

**General Anesthesia/Malignant Hyperthermia Checklist**

_____Administer Medications to get patient to sleep (general anesthesia)

_____Perform Endotracheal Intubation

_____Recognize Intraoperative emergency

_____Stop surgery/Turn off Anesthetic Gases

_____Work together to find and administer dantrolene

**Opioid Overdose Checklist**

_______Recognize that the patient is unresponsive and in respiratory failure due to opioid overdose

_______Appropriately treat the patient with naloxone

_______Recognize the progressive dyspnea after the naloxone administration

_______Reassess the patient and identify pulmonary edema

_______Attempt to oxygenate and ventilate the patient using either a non-rebreather oxygen mask or other non-invasive methods.

_______Recognize that the patient’s O2% saturation is not improving by any means, and perform intubation.

_______Establish /maximize lung ventilator settings

_______Recognize that oxygenation is still poor despite maximizing ventilator settings

______ Get a consult for ECMO or other advanced management

**Stroke/Neurological Assessment Checklist**

______Identifies self to patient/patient family

______takes history to establish risk factors

______Takes history to establish time course of symptoms

______ complete neurological assessment of patient with cerebrovascular disease w/NIH Stroke Scale

______Identifies and suggests treatment for hypertension

______Identifies need to give TPA

**Heart Attack/Cardiac Arrest Checklist**

_____Demonstrates an approach to a patient with chest pain

_____Team works well together

_____Recognizes and addresses hypotension

_____Intubation

_____Effectively recognizes and addresses cardiac arrest
