# Supplementary material for: Advancing health equity through simulation-based education for pre-health students
Source: PLoS One. 2026 Aug 12;21(8):e0354377. doi: 10.1371/journal.pone.0354377 (PMC13465807; doi:10.1371/journal.pone.0354377)
Supplement: Supplementary file 2 [file pone.0354377.s002.docx]

| Simulation Case  SIMULATION CASE TITLE: General Anesthesia & Malignant Hyperthermia: An Anesthesiology Simulation Case | |
| --- | --- |
| PATIENT NAME:  PATIENT AGE:  CHIEF COMPLAINT: Undergoing elective inguinal hernia repair surgery under general anesthesia | |
|  | |
| Brief narrative description of case | The patient _________ is a 40-kg __-year-old Caucasian male who is undergoing an elective inguinal hernia repair. His past medical history includes asthma. His past surgical history includes circumcision at birth. He was brought to the operating room with a working 20g IV in the hand.  Learner goals:  Identify and treat malignant hyperthermia (MH) in the intraoperative setting. |
| Primary Learning Objectives | A. Understand components of routine GA with ETT placement. B. Present a case of MH so that participants can recognize the signs and symptoms. C. Discuss differential diagnosis of malignant hyperthermia. D. Identify and understand the chronological order of developing MH, which includes:    i. End-tidal carbon dioxide    ii. Heart rate    iii. Temperature    iv. Muscle rigidity E. Identify the change in blood tests:    i. Arterial blood gas    ii. Electrolyte panel    iii. Renal function F. Apply the algorithm for treatment of MH using the Malignant Hyperthermia Association of the United States (MHAUS) Emergency Therapy Protocol, including dantrolene administration, with emphasis on teamwork. G. Recognize patient’s response to appropriate (and/or inappropriate) management interventions. |
| Critical Actions | 1. Alert the OR staff/Call for help 2. Call for the MH cart 3. Turn off anesthetic volatile agents and increase fresh gas flows 4. Use activated charcoal filters (Vapor-Clean™, Dynasthetics, Salt Lake City, UT) 5. Create an organized method with team members to administer dantrolene 6. Continue supportive care to keep patient hemodynamically stable and normothermic |
| Learner Preparation | Learners should have reviewed the triggering factors, signs and symptoms, and treatment protocol of malignant hyperthermia in the operating room setting. |

| Initial Presentation | | | |
| --- | --- | --- | --- |
| Initial vital signs | HR 80, BP 115/75, Temp(C) 37, etCO2 38, SaO2 99%, RR 11, WT 40-kg | | |
| Overall Appearance | The patient is awake with a peripheral 20g IV with normal saline. The surgical drape is attached to two IV poles, and the surgeon is operating in the inguinal region on the patient. The anesthesia machine and cart are at the head of the bed and a vital sign monitor is displaying the patient’s heart rate, EKG, blood pressure and pulse oximeter. | | |
| Actors and roles in the room at case start | Simulation Learners (at least 2 individuals in an anesthesia provider role)  Nursing Staff (Scrub Nurse/Circulator; these roles may be performed by a single actor)  Surgeon | | |
| HPI | The patient _____is a 40-kg ___-year-old ____ male who is undergoing an elective inguinal hernia repair. His past medical history includes asthma, with rare attacks occurring during winter months. He had been circumcised at birth, and has no family history of anesthesia related complications. He is an active child, and eats a healthy, well balanced diet. In the preoperative phase, he was given oral midazolam and a 20g IV in the right hand was placed without difficulty. He was then brought to the operating room. and an IV induction was performed with propofol, fentanyl, and succinylcholine.  The role of the learner is to administer medications, intubate, manage the anesthetic requirements and monitor the vital signs during the operation. The learner should identify any abnormalities consistent with malignant hyperthermia and treat them accordingly during this time. | | |
| Past Medical/Surgical History | Medications | Allergies | Family History |
| Asthma, controlled with less than 2 attacks/month, triggered by cold weather  Surgical history: circumcision | Albuterol inhaler for wheezing/bronchospasm | none | parents deny any history of adverse events from anesthesia |
| Physical Examination | | | |
| General | Active, responsive | | |
| HEENT | WNL, Mallampati Class I, full dentition, full neck ROM; TM distance 5 cm | | |
| Neck | WNL; full range of motion | | |
| Lungs | WNL | | |
| Cardiovascular | Regular rate and rhythm, no murmurs | | |
| Abdomen | WNL | | |
| Neurological | Patient is awake, responsive, sleepy | | |
| Skin | WNL | | |

| Instructor Notes - Changes and CASE Branch Points | | |
| --- | --- | --- |
| Intervention / Time point | Change in Case | Additional Information |
| 3 minutes into the case | Instructor will inform the learner 15 minutes have passed since incision. Vital signs now show HR 105, BP 132/90, Temp 37.7 C, ETCO2 45, SaO2 98%, and RR 16, with muscle rigidity on physical exam |  |
| 1-2 minutes after first vital signs changes | Vital signs gradually increase to HR 120, BP 149/101, Temp 38.4 C, ETCO 54, SaO2 95%, RR 25, and surgeon mentions muscle rigidity in the surgical field. | Instructor will point out vital sign changes if not apparent to learners and ask how the learner plans to respond to the new changes. |
| 5-8 minutes after initial change in vital signs |  | Learner should already suspect MH. If learners have not reached this possibility, instructor inform them of the likely diagnosis of malignant hyperthermia |
| Facilitator requests an ABG and other labs | ABG shows pH 7.25, pCO2 55, pO2 95, HCO3 16. |  |
| Learner alerts OR staff to high suspicion of MH and calls for help and for the MH cart to be brought into room | MH cart is brought into room, and additional staff arrives at room. | Instructor has one learner become leader for organizing roles in MH protocol and preparation of dantrolene. |
| Learner should turn off all volatile agents and administer 100% FiO2 |  | Instructor should remind learners if not already performed. |
| Dantrolene is administered to patient at 2.5mg/kg | Vital signs should start to stabilize and return to normal values. |  |
| Additional IV access should be discussed |  |  |

Ideal Scenario Flow

The learners enter the room to a pediatric patient who has already received midazolam. Vital signs are displayed on a monitor, including heart rate, blood pressure, and pulse ox. Learners need to administer medications (propofol and fentanyl), get the patient off to sleep (administer succinylcholine) and orally intubate patient under direct laryngoscopy with a MAC 3 blade, 6.0 mm ID cuffed endotracheal tube placed and secured with tape. Additional ventilator data and end-tidal CO2 should also be on display. Volatile anesthetics on. The scrub tech has just finished setting up the sterile field, and the procedure is about to begin. A surgeon is operating on the inguinal area. Initial vital signs are normal and stable. The surgeon proceeds with the surgery and after the instructor announces 15 minutes have passed since incision, the learners will notice the ETCO2 gradually rising and heart rate increasing. They immediately recognize malignant hyperthermia a likely diagnosis. Facilitator orders ABG. Learners alert the OR staff, and request the surgeon to stop the surgery at a non-critical point. The learners will turn the volatile anesthetic off, start delivery 100% FiO2, request the MH cart be brought to the room and have the OR staff start preparing dantrolene. They will administer the dantrolene at 2.5mg/kg and continue monitoring the patient for signs of improvement. Once stable, the patient is sent to the ICU for further supportive care with serial blood gases and urine measurements to be monitored for resolution of malignant hyperthermia.

Anticipated Management Mistakes

1. Failure to recognize the diagnosis of MH: We realized that many learners did not immediately arrive at the possible diagnosis of malignant hyperthermia. If this happened, the instructor would have the vital signs changed to a worsening stage of MH with temperature rising with tachycardia and hypercarbia and the surgeon would note muscle rigidity in the surgical field. If the learners still do not realize the diagnosis, the instructor will explain the patient is expressing signs and symptoms consistent with MH and ask the learner for the next step in management.
2. Failure to recognize the need for the MH cart and dantrolene: Some of learners were unaware of the need to treat MH with dantrolene and that the MH cart would be necessary to prepare and administer the treatment. If this occurred, the instructor would help guide the learners to organize the OR staff and call for the cart to prepare the treatment.
3. Failure to recognize the need to turn off volatile anesthetics: Some learners did not recognize the need to turn the offending agent, the volatile anesthetic, off and administer 100% FiO2. If this occurred, the patient would continue to worsen until the learner recognized this step or the instructor intervened and informed the learner to turn the agent off.

| **Appendix A: MedEdPORTAL Simulation Case**  **SIMULATION CASE TITLE: Opioid overdose**  **AUTHORS: Michael P Keenan, BS, Keith A Schenker, MD, Matthew J. Sarsfield, MD** | |
| --- | --- |
| **PATIENT NAME:**  **PATIENT AGE:**  **CHIEF COMPLAINT: Unresponsive** | |
|  | |
| **Brief narrative description of case** | This case describes a __-year-old male patient presenting unresponsive after an opioid overdose. He responds well to naloxone, but he quickly develops dyspnea secondary to flash pulmonary edema. The learner goals are to recognize this potential complication of naloxone and to appropriately treat it. |
| **Primary Learning Objectives** | - Describe mechanism of opioid induced pulmonary edema and naloxone induced pulmonary edema - Execute appropriate pharmacological and airway management of an opioid overdose patient using equipment available - Execute the best management for a patient in flash pulmonary edema that is difficult to oxygenate - Demonstrate team work and communication skills by: - Appointing a team leader - Team leader assigns clear roles and tasks to available team members - Team utilizes closed-loop communication as appropriate - Team communicates in an open and respectful manner |
| **Critical Actions** | 1. Recognize that the patient is unresponsive and in respiratory failure due to opioid overdose 2. Appropriately treat the patient with naloxone 3. Recognize the progressive dyspnea after the naloxone administration 4. Reassess the patient and identify pulmonary edema 5. Attempt to oxygenate and ventilate the patient using either a non-rebreather oxygen mask or NIPPV. 6. Recognize that the patient’s O2% saturation is not improving by any means, and perform intubation. 7. Recognize that oxygenation is still poor despite maximizing ventilator settings 8. Initiate advanced ICU management & ECMO consult |
| **Learner Preparation** | The patient is a __ year-old male who presents to the ER by EMS after he was found unresponsive in a nearby park by his friends. He is only producing agonal respirations. |

| Initial Presentation | | | |
| --- | --- | --- | --- |
| **Initial vital signs** | T 37.4oC, BP 110/75, HR 110, RR: agonal, O2% Sat: 65% | | |
| **Overall Appearance** | Patient is unresponsive with agonal respirations | | |
| **Standardized patient (SP) and roles in the room at case start** | No SP | | |
| **HPI** | EMS informs the learners that the patient was found unresponsive. His friends informed the EMS personnel that before going unresponsive, the patient had injected heroin. EMS administered 0.4 mg of naloxone on the ambulance during the 5-minute transport time before arrival without any effect. | | |
| **Past Medical/Surgical History** | **Medications** | **Allergies** | **Family History** |
| Asthma | Albuterol inhaler | None | None |
| **Physical Examination** | | | |
| **General** | Patient is initially unresponsive, but appears dyspneic after revival with naloxone | | |
| **HEENT** | Pupils are initially 2 mm and minimally reactively. They dilate to 4 mm after naloxone administration and are reactive. | | |
| **Neck** | No pertinent findings | | |
| **Lungs** | Initially, patient’s respiratory rate is profoundly low. Lung sounds are equal bilaterally without wheezes, rales, rhonchi. After administration of naloxone, patient’s respiratory rate increases and he becomes dyspneic. Reevaluation demonstrates bilaterally rales on auscultation. | | |
| **Cardiovascular** | Regular tachycardic rhythm | | |
| **Abdomen** | Soft and non-tender on exam. | | |
| **Neurological** | GCS 3 on arrival. GCS 15 after appropriate naloxone administration. Eyes open, confused and mumbling, not following commands. GCS 13 | | |
| **Skin** | No pertinent findings | | |
| **GU** | No pertinent findings | | |
| **Psychiatric** | No Pertinent findings | | |

| Instructor Notes - Changes and CASE Branch Points | | |
| --- | --- | --- |
| **Intervention / Time point** | **Change in Case** | **Additional Information** |
| *Naloxone given* | *Patient rapidly responds to naloxone. GCS increases to 13.*  *New vitals: BP 138/72, HR 124, RR 45, O2% Sat 52% NRB, Temp 36.5oC* | *GCS – 2 points for inability to follow commands (localizes painful stimuli) and confusion* |
| *Learners reassess the patient* | *Patient responds to learner questions and provides history described above (Manekin)* |  |
| *5 minutes after naloxone is given* | *Patient becomes progressively dyspneic, respiratory rate increases, O2% sat drops to 60%* | *Patient may state “doc it’s getting really hard to breath”* |
| *5 minutes after patient develops dyspnea* | *If no attempt has been made to improve the patient’s oxygenation, the patient’s vitals begin to get progressively worse* | *A “nurse” (the SP for the patient’s voice) should enter the room briefly and ask the learners if they want to do anything for the patient’s vitals* |
| *Learners reassess patient* | *Rales are present on physical exam* | *If learners ask for CXR at any point after this, a CXR is provided that demonstrates bilateral pulmonary edema* |
| *Learners may attempt duo-neb or albuterol based on the history above* | *No change* | *Patient may state “doc I’m really struggling here. What’s happening?”* |
| *Learner may attempt NIPPV* | *No change* | *Patient struggles against the mask and constantly removes it* |
| *Either after patient has attempted one of the above interventions or after 5 minutes* | *Patient begins to deteriorate (RR decreases to 8, O2% Sat 60%, GCS 7)* |  |
| *RSI Intubation or awake intubation* | *If successful: No change to O2% sat*  *If unsuccessful: Patient’s vitals drop precipitously* |  |
| *Learner should set ventilator to lung protective settings* | *No change* |  |
| *Learner should recognize need for ECMO* | *Case ends* | *If ECMO is performed by the emergency physicians at the learner’s institution, they can set it up based on local protocol. If it is not, they must recognize*  *the need and make appropriate consults* |

Ideal Scenario Flow

Learners discover from EMS that the patient was found unresponsive after injecting heroin. They should administer naloxone to reverse the opioid overdose. The patient will respond to the antidote and will regain consciousness. The learner should complete a history and physical exam. However, the patient develops progressive dyspnea and his O2% saturation holds in the low 60s%. The learner should recognize this and reassess the patient. They should discover rales on exam. The learners should order a chest x-ray at some point, which will show pulmonary edema. They may attempt simple oxygen administration or noninvasive positive pressure ventilation to improve oxygenation. However, there will be no improvement and the patient will gradually decline. The learner should recognize the need for intubation. Oxygenation does not improve, despite intubation. The learner should recognize the need for ECMO in this patient and call the respective team responsive for ECMO.

Anticipated Management Mistakes

1. Failure to recognize failure of the patient to oxygenate: Learners may not recognize that the patient is dyspneic and that their O2% sat is low after naloxone administration. If they do not notice, a “nurse” can enter and ask if the learners want to do anything for the patient’s vitals. The SP can also further exacerbate his dyspnea.
2. Failure to recognize need for ECMO or advanced ICU management: Learners may not recognize the need for ECMO or advanced ICU management in this patient, especially if they have not encountered a patient like this before. If the learners are struggling, the case can be stopped at this point and management options can be discussed further in the debrief.

| Date:  Discipline: Interdisciplinary  Expected Simulation Run Time: 20 min  Location: Emergency Room  Today’s Date: | File Name:  Student Level: college students learning about stroke, neurological assessment  Guided Reflection Time: 20 minutes |
| --- | --- |

Psychomotor Skills Required of Participants

Neurologic assessment

Cognitive Activities Required of Participants

- Pathophysiology of strokes caused by a clot
- Management of stroke related to physical changes and motor ability
- Psychosocial and neurologic assessment

Simulation Scenario Objectives

1. Complete a neurological assessment of a patient who has had a stroke.
2. Become familiar with interventions for a stroke patient.

Simulated Patient/Manikin(s) Needed: Use of a SP with disability is ideal for authenticity of the experience. Another SP or faculty member who is educated to simulate the disability is the alternative. Simulate the disability by having right-sided weakness and inability to use right arm and right leg. You have sensation, but cannot move those extremities. You use left arm to move right arm. When walking you swing your right leg as you walk (as if it is stiff).

Other Props & Moulage: Have a printed set of vital signs available in the room, as if taken by tech or UAP (on a clipboard or in large print hanging in the room).

Pre-briefing/Briefing

Prior to report, participants will need pre-briefing/briefing. During this time, faculty/facilitators should establish a safe container for learning, discuss the fiction contract and confidentiality, and orient participants to the environment, roles, time allotment, and objectives.

# **Case Title / Topic: TPA Stroke Case**

## Learning Objectives or Assessment Objectives

After completing this simulation learners will be able to

1. Distinguish stroke from stroke mimics based on clinical features

2. Perform a neurologic exam using NIH Stroke Scale

3. Gather relevant information (historical, clinical, laboratory, imaging)

A. Critical actions checklist:
1. Identifies self to patient / family
2. Asks about time of symptom onset
3. Asks about history of presentation
4. Asks for finger stick glucose value
5. Asks about risk factors in the PMH (relevant history of diabetes, hypertension, hyperlipidemia, atrial fibrillation, coronary artery disease, history of previous strokes etc.)
6. Requests appropriate labs (CBC, BMP, Troponin, INR) immediately
7. Performs focused exam (completes NIHSS)

• Alert

• Orientation (time, place, person, situation)

• Pupillary reaction

• Facial symmetry (smile, frown, raise eyebrows, etc.)

• Bilateral grip strength

• R & L Arm resistance to pressure

• R & L Leg resistance to pressure

• May also check for pressure points r/t immobility

8. Gives 10 mg labetalol for SBP > 185
9. Reviews lab results
10. (Optional) Reviews CT. Can also be done by phone call/written report, etc. by neuroradiologist. CTA optional for institutions who have capabilities of either in house access to interventional vascular services or can timely transfer to such.
11. Reviews ECG /rhythm strip.
12. Orders TPA

15. Requests for hospital admission

16. Delegates effectively to team members:

- Data collection
- Collateral history
- Verification of time of symptom onset

## IV. Environment

A. Venue Set Up
1. Type of Room / Area: ED room / critical care bay
2. Beds / stretchers: usual ED stretcher
3. Other large equipment: Cardiac/BP monitor, crash cart

```
        Electronic/computer screen

B.  	Supplies
```

1. IVs & Fluids Needed: IV -18-gauge which will be taped on standardized patient, not inserted. This will be “placed” prior to the neurology resident arriving.
   a. EKG leads for cardiac monitoring
2. Drugs needed (empty vials with labels on, nurse confederate will act as if administering when prompted but no drug actually given to the actors)
   a. Hydralazine
   b. Labetalol
   c. Nicardipine
   d. TPA
   e. Atropine
   f. Dopamine / Dobutamine
   g. D50
3. Other Medical Equipment Needed:
   a. EKG lead tracing
   b. Lab result print out for basic chemistry, glucose, Hgb. Most commonly basic labs would be sent out by ED resident, before calling stroke code so it is implied that during the time when resident runs the stroke code they should be back upon his/ her asking/ensuring the labs. (Only glucose is necessary before TPA administration but in this case INR would be critical as well since patient is on warfarin. INR will not return during the case, but if participants ask to do a chart review, they find that the INR was 1.4 earlier that morning from a clinic visit. (INR results are critical as cannot administer tpa if INR is not <1.7.)

C	Props

1. EKGs: Regular sinus rhythm, also BP results on patient monitor
2. CT scans: CT head, CTA head and neck (shows thrombus in Left MCA) but no cerebral hypodensity involving greater than 1/3 territory of MCA territory
3. Lab results in a printable / electronic format to show participants
   a. Chemistry is normal, aside from glucose 45
   b. Hgb normal
   c. INR 1.4
4. Hospital chart information or other materials (*basic EMS run sheet – does not have a fingerstick blood glucose listed or performed)*

## V. Actors

A. Roles
1. ED Nurse
2. Patient
3. Family member
4. Neurology faculty
B. Who may play them (*ie. trained actors, other volunteers)*
   1. ED Nurse – any well trained actor or ED / ICU nurse
   2. Patient – any well trained actor
   3. Family Member – any well trained actor
   4. Neurology faculty (over phone only) – played by neurology faculty, EM faculty or neurology senior resident
C. Action Role
   1. ED Nurse – nurse’s competence will be inversely proportional to the learner’s competence
   2. Patient – aphasic but cooperative
   3. Family Member – as per instructions below in play of case guidelines. Overall cooperative and provides necessary information when asked.
   4. Neurology faculty (over phone only) – only is used if learners require additional assistance in the case and cannot be directed well enough with nurse confederate prompts.
D. Participants of simulation
1. Emergency Medicine professional (learner initially assessing patient)
2. Neurology specialists in groups of 3 (one running stroke code and other 2 acting as team members), arrive once stroke code is called
3. Additional learner team members when desired.
VI. Case Information
A. Case Narrative: Scenario background given to participants (scene introduction should be given freely but additional details only revealed if asked for)
1. Scene introduction for learner: You are working in the critical care bay; you have been informed that a new patient is on the way and will be arriving shortly, no further information available.
2. Scene introduction for Neurology resident: You are rounding at your hospital as G2 resident with your attending and your pager goes off indicating a stroke code. You are about to enter the critical care bay in the ED.
3. Case Synopsis (to be provided to moderator and standardized patient, family member and nurse):

_________ is a ______year old ______ who was last seen normal at X pm (time should be set to fall within 2 hours of stroke code simulation) by his son (or daughter, depending on available confederate). Son went out for 10 minutes, when he came back, he saw ________ lying on the floor with unintelligible speech and unable to move his right side. Son immediately calls 911 and patient is brought to the ED. Patient came within 2 hours of time of symptoms onset.

ED resident first assesses the patient and then stroke code should be activated at which time neurology resident joins the simulation.

Neurology resident with his/her team (total of 3) enters the room, examines the patient, and performs the required assessment, formulates the plan based on exam, labs, imaging and talking to family members.

The confederate nurse should provide lab results when asked for by any of the participating learners. Neurology specialist over phone should decide to give TPA in light of INR < 1.7 and indicates intention to discuss the case with interventional team.

This encounter is closed after 20 minutes.

4. Chief complaint: Cannot talk/speech is garbled, is not moving right side.
5. Past medical history: Hypertension, Paroxysmal atrial fibrillation, Chronic renal failure, Congestive cardiac failure, Diabetes. No contraindications to tPA.
6. Medications: Warfarin, Digoxin, Diltiazem, Atenolol, Lipitor, Insulin (lantus and aspart)
7. Allergies: NKA
8. Family history: no history of stroke or vascular risk factors in past
B. Scenario conditions initially
1. History patient (or family, parent, etc) gives: Since patient cannot talk family member will provide history, details mentioned in above case synopsis.
2. Patients initial exam: (following deficits will be acted out by patient.
   a. VS: BP 190/85	HR	88	RR	15	SpO2	98%	T 37.4
   b. General:
      i. HEENT: normal, PERRL
      ii. Neck: supple, FROM
      iii. CV: No m/r/g
      iv. Pulm: CTAB
      v. Abd: normal
      vi. Extremities: no trauma, no c/c/e
      vii. Skin: no rash
   c. Neuro (the following deficits will be acted out by patient): Weakness of right face and right arm more than leg. Garbled unintelligible speech, unable to read aloud and write if asked to.
C. Scenario branch points:
1. If no initial self-identification, he or she is to be ignored by patient and family member. Family member will continue to address and talk to the nurse, and be dismissive of questions asked by the learner. If no self-identification by the resident lead 2-3 minutes into the case, the family member will ask the learner “who are you?”. Once introduces themselves to the family member, they will be cooperative and conversant.
2. If stroke code not called by the EM professional within 5 minutes of case starting, the nurse asks “what do you think is going on here?”
a. If EM professional does not say stroke or state that they will page the stroke team, then nurse asks why the patient can’t move his right side.
b. Nurse gives progressively stronger prompts to resident until nurse suggests to call the stroke team if the EM resident is not getting to that endpoint.
3. If no time of onset clarified within 5 minutes of neurology resident arriving to case, nurse prompts and asks the patient / family member what time the symptoms started.
4. If glucose checked, nurse will report that fingerstick glucose is 45.
5. If glucose not checked, allow the case to proceed. If not self corrected, patient would receive tpa and provider will learn that patient was hypoglycemic only after BMP comes back (Glucose 45)
6. If D50 is given or not given, neuro exam does not change.
7. BP continues to rise over first few minutes of the case regardless of learner actions. BP is cycled at 2 minutes into the case and reads 206/95.
a. If BP not treated, BP continues to rise to 250/130 over first 8 minutes of the case.
b. If one dose of =<20mg labetalol given, no change in BP
c. If total of 30-50 mg labetalol given, BP goes to 165/72
d. If total of 51-80mg labetalol give, BP drops to 124/62
e. If hydralazine, nicardipine, or other antihypertensives are given, the appropriate starting dose does produce desired effects of BP < 185, and a second dose or increase in dose is required to meet this endpoint. If excessive doses of any antihypertensives are given, BP with precipitously drop as in labetalol dosing instructions above.
8. If participants discuss tPA administration with the patient’s family member, the family member is easily agreeable to this plan.
9. If participants do not discuss tPA with the family member within 10 minutes of neurology resident arriving to the case, THEN
a. Family member asks “isn’t there anything you can do to help my dad?”
b. If no response from resident regarding tPA, family member states “can you give him some sort of medication or something to help him?”
c. If resident still does not discuss tPA, neurology faculty calls in and asks resident about the patient including their thoughts on the management plan, and gives some guidance / suggestions if needed. Neurology faculty will also call in if participants are having difficulty with management of other aspects of the case after 15 minutes has elapsed in the case.
10. If INR is sent, this will not return during the case.
a. If participants already asked nurse to send INR prior to asking, s/he will state “the result isn’t back yet, I sent the tubes though.”
b. If the participants did not ask the nurse to send INR prior to asking, s/he will state “oh, did you want me to send that? I can do it now.”
11. If participants ask to look in the patient’s chart to see his last INR, the nurse can look in the chart and report that the INR was 1.4 this morning. If participants do not ask to do chart review, the case will continue, and this will be discussed in the debriefing.
12. If INR is not ordered or asked about, there is red fluid in foley following tPA administration (nurse trained to prompt)
VII. Instructors Notes (what the instructor must do to create the experience)
A. Dry run with involved actors, debriefer and staff for identification and troubleshooting of any potential loopholes

#### B. Orientation of actors about the case synopsis

C. Scenario programming
1. Optimal management path

```
 Appropriate delegation of tasks to various members of stroke team, stab room door to tPA order time (the time from start of stroke team evaluation in stabilization/ED room to actually ordering tPA) should be  10-15 minutes
		2.	Potential errors path – see above in play of case guidelines
```

## VIII. Debriefing Plan

A. Method of debriefing –*group debriefing (for all the participants)*
B. Actual debriefing materials (include NIHSS hand out, and Acute stroke management Hand out).
C. Questions to facilitate the debriefing (*these are examples below – feel free to use some / all / none of these or design your own*)
1. So, how do you feel about this case? (Reactions Phase)
2. When you initially encountered the patient who was unable to respond to questions appropriately, what was going through your mind? (Understanding Phase)
3. What was going through your mind when the low blood sugar was discovered? (Understanding Phase)
4. Do you have an approach that you use every time for possible stroke cases? (Understanding Phase)
5. What main learning points will you take away from this case and apply in the future? (Summary Phase) (I often go around the table and have each participant state one learning point, and then add on any important take home points that were not restated during this activity at the end.)

## IX. Pilot Testing and Revisions

A. Numbers of participants: maximum of 3 per case

Implementation:

We organized a stroke simulation day that consisted of four stations dedicated to different aspects of acute stroke care. Two of the stations were high fidelity simulations in the setting of an emergency room and the other two of the stations were skill stations that were dedicated to JHACO comprehensive stroke center requirements as well as intricacies of the NIHSS in difficult situations. This submission is only covering one high fidelity case.

Section 1: Case Summary Scenario

Title: Rural STEMI

Brief Description of Case: Inferior STEMI in a community hospital.

Goals and Objectives:

1. Develop an approach to a patient with chest pain.

2. Communicate effectively with team members in the care of a complex, critically ill patient.

3. Recognize and manage evolving chest pain and cardiogenic shock.

4. Develop an approach to intubation in a patient with cardiogenic shock.

5. Understand the protocol for addressing cardiac arrest

Recommended Number of Facilitators:

Instructors: One to two Confederates: One Sim Techs: One as needed if mannequin available

The scenario is run with the help of a confederate, an individual who, during the course of the scenario, provides assistance with equipment, provides information about the mannequin not available in other ways (e.g. temperature, colour change), and/or to provide additional realism by playing the role of a relative or staff member.

Three to four learners participate in the scenario, one as a team leader and the others in additional roles. If the scenario is run with only one learner, the learner is to assume the role of team leader and available confederates can be used as the additional team members.

Personnel

Two facilitators, who were familiar with the scenario and its management, were present during the scenario. One ran the scenario, took notes, and provided prompts as needed. The other acted as a nurse confederate assisting with the scenario in order to meet the learning objectives. They performed tasks for the learners as requested and delivered results and prompts to the learners when needed. When fewer residents are available to partake in the simulation, more confederates may be used to assist the learners.

Equipment

A Laerdal SimMan 3G® human patient simulator. The equipment included the following items:

- Advanced cardiac life support defibrillator and standard medications

- Airway supplies including oxygenation, intubation supplies, and suction

- Hospital chart and electrocardiograms (EKG)

- Intravenous (IV) access supplies and fluids

- Cardiac and oxygen monitors

- Medications, including vasopressors and inotropes

Step-wise, detailed scenario template.

BIPAP – bilevel positive airway pressure; BP – blood pressure; bpm – beats per minute; CPR – cardiopulmonary resuscitation; CXR – chest radiograph; EKG – electrocardiogram; HR – heart rate; ICU – intensive care unit; IV – intravenous; min – minutes; Nitro – nitrogylcerin; NRB – non-rebreather; Q – every; RA – room air; RR – respiratory rate; RT – respiratory therapist; SL – sublingual; SOB – short of breath; SpO2 – oxygen saturation; T – temperature; VBG – venous blood gas; VT – ventricular tachycardia

| STATE | STATUS | LEARNER ACTIONS | OPERATOR NOTES |
| --- | --- | --- | --- |
| 1. Nurse call | Nurse calls or presents with chart. Shows most recent EKG (Figure [1](https://pmc.ncbi.nlm.nih.gov/articles/PMC7388803/table/TAB1/#FIG1)) and does not provide vitals. | Ask nurse for vitals |  |
|  |  | Ask nurse for previous EKG |  |
|  |  | As nurse for chart |  |
|  |  | Instruct nurse to place patient on monitors |  |
|  |  | Go see patient |  |
| 2. Initial assessment | T: 36.7 °C | Get IV access | Modifiers: |
|  | HR: 101 bpm | Apply oxygen | No repeat EKG ￫ Nurse to cue |
|  | BP: 181/94 | Draw bloodwork including VBG and troponin | Patient to cue still having chest pain after each spray of nitro |
|  | RR: 28 | EKG | Triggers: |
|  | SpO2: 91% RA | Read through chart | Nitro  ×3 ￫ 3. Deterioration |
|  | Patient alert and able to answer questions. Diaphoretic | Nitro spray 0.4 mg SL Q5min ×3 | After 5 min nurse to cue patient looks more SOB ￫ 3. Deterioration |
| 3. Deterioration | T: 36.7 °C | Trial 250-500 mL crystalloid bolus | Modifiers: |
|  | HR: 110 bpm | Repeat EKG, can consider 15 lead | Bolus given ￫ BP 90/50 |
|  | BP: 84/40 | Capillary sugar | After 5 min, BP and SpO2 continue to trend down |
|  | RR: 30 | Prepare for intubation | Triggers: |
|  | SpO2: 85% on 100% NRB |  | Prepare for intubation ￫ 4. Intubation |
|  | Patient confused. Cool, clammy. |  | No intubation prep ￫ nurse to cue calling RT ￫ 4. Intubation |
| 4. Intubation | As above | Consider BiPAP | Modifiers: |
|  |  | Pre-medicate with fentanyl | Propofol used or unsafe intubation ￫ 5. VF arrest |
|  |  | Induce with ketamine | Triggers: |
|  |  | Paralytic used | Intubation successful ￫ 6. Ongoing hypotension |
|  |  | Vasopressor at bedside |  |
| 5. VF arrest | Patient pulseless and unresponsive. | High quality CPR | Triggers: |
|  |  | Defibrillate | 2 cycles CPR ￫ 6. Ongoing hypotension |
|  |  | Epinephrine 1 mg IV Q3min |  |
|  |  | Amiodarone 300 mg IV |  |
| 6. Ongoing hypotension | T: 36.7 °C | CXR (Figure [2](https://pmc.ncbi.nlm.nih.gov/articles/PMC7388803/table/TAB1/#FIG2)) | Modifiers: |
|  | HR: 120 bpm | Sedation | Norepinephrine started ￫ BP 70/40 |
|  | BP: 65/30 | Heparin | Norepinephrine titrated up 2-3× ￫ BP 80/45 |
|  | SpO2: 92% on 100% O2 | Start norepinephrine infusion and titrate up | Dobutamine added ￫ BP 90/50 |
|  |  | Consider another 500 mL crystalloid bolus | Triggers: |
|  |  | Consider starting dobutamine | Vasopressor and inotrope started ￫ 7. Stabilization |
|  |  |  | Consider consult cardiology/ICU ￫ 7. Stabilization |
| 7. Stabilization | As above. | Finish tasks from above | END OF SCENARIO |
|  |  | Consult cardiology if not done |  |

Pre-scenario Information

You are a resident working overnight in the emergency department of your community hospital. A nurse from the inpatient ward comes to your office with an EKG from a patient who was admitted earlier in the day and is currently complaining of chest pain.

Initial Patient Information

A. Patient Chart

Patient Name: ______ Age: ___ Gender: ______ Weight: 100 kg

Presenting complaint: Chest pain continuous for last 1 hour, intermittent exertional chest pain over last 2 weeks

Temp: 36.6 HR: 95 BP: 175/80 RR: 16 O2Sat: 96% FiO2: RA Cap glucose: 9.0

GCS: (E V M ) 15 (4,5,6) Triage note: Central squeezing chest pain constant for last 2 hours, intermittent over last 2 weeks with exertion. Mild nausea, no vomiting. Allergies: NKDA Past Medical History: HTN DM2 GERD

Current Medications: Ramipril 5mg po daily Metformin 1g po BID Pantoprazole 40mg po daily

Section 2B: Extra Patient Information

A. Further History History provided by the patient - The chest pain is central, squeezing, non pleuritic and radiating to her jaw and left shoulder. Severity is 7/10. She has mild nausea with the pain, but has not vomited. She has no back pain, no sob, no cough, no fever, no leg swelling. She has a history of GERD but this is nothing like that. The pain came on while she was sitting on the couch watching TV and hasn’t gone away. She has been getting pain like this over the last 2 weeks with exertion but it would resolve with rest in the past, which is different from this episode.

PMHx: as above; PSHx: Cholecystectomy 2y ago;

Social Hx: No ETOH, 10py smoking hx, quit 20y ago, no drugs

FHx: 2 Brothers and her father have had heart attacks, father died of a stroke at 65.

B. Physical Exam List any pertinent positive and negative findings

Cardio: NS1S2. No extra heart sounds, no murmurs

Neuro: Grossly normal. No focal deficits.

Resp: GAEB No increased work of breathing. Head & Neck: JVP 3 cm above sternal angle. Otherwise normal.

Abdo: Soft, non-tender, no masses, no organomegaly.

MSK/skin: Normal.

Other: Mildly diaphoretic. Pain not reproducible with palpation or movement

EKGs and imaging:

https://litfl.com/wp-content/uploads/2018/08/ECG-Right-ventricular-infarction-1.jpg

Technical Requirements/Room Vision

A. Patient ☒ Mannequin Adult, if available ☐ Task Trainer ☐ Hybrid

B. Special Equipment Required In situ simulation utilizing participants’ usual facilities and equipment. Mannequin if available. Interior Health STEMI PPO.

C. Required Medications Whatever is available on site. SIM will utilize: Nitro, Morphine, ASA, Clopidogrel, IV fluids, Oxygen, Ramipril, Atorvastatin, Metoprolol, TNK (tenecteplase).

D. Moulage None

E. Monitors at Case Onset ☒ Patient not yet on monitor

F. Patient Reactions and Exam Include any relevant physical exam findings that require mannequin programming or cues from patient (e.g. – abnormal breath sounds, moaning when RUQ palpated, etc.) Initial vitals on mannequin to show SpO2 96% on room air, HR 95, NSR, BP 175/80, RR 16.

The content of the debriefing session guided by the case learning objectives, yet it was a fluid process to accommodate the evolving dynamics of the team behavior. That is, depending on how the scenario evolves, modifications were made to address any learning needs that arise. In this session, residents were taught around what to expect clinically from each infarct territory, the importance of serial EKGs, and the management of cardiogenic shock in the context of an acute MI. Considerations, such as the use of small boluses, vasopressor initiation, and when to consult a specialty service were also covered. The use and contraindications of TNK and intubation techniques were discussed. An approach to chest pain, including a differential diagnosis of non-cardiac causes of chest pain was also discussed. Additional objectives may be included by individual facilitators depending on local goals of the session. In cases such as this where transfer may be required, there is an opportunity to discuss local protocols and considerations such as weather and resource management.

Modifiers for the case can be applied in a variety of ways to both adjust difficulty and allow repeated use of the case, highlighting different points at each encounter. One such modification could include death as the end scenario. Depending on learner objectives, facilitators could include an unsuccessful resuscitation as an optional end to the case.

Locally-developed global rating scale.

ACS - acute coronary syndrome

|  | Clearly below expectations |  | Acceptable performance |  | Performs above expectations |
| --- | --- | --- | --- | --- | --- |
| Initial management of a patient with chest pain | 1 | 2 | 3 | 4 | 5 |
|  | Demonstrates deficient knowledge of acute cardiac presentations without recognizing and correcting it. | Weak knowledge of acute cardiac presentations. Made errors but did not recognize or correct them. | Competent performance that indicates working knowledge of ACS but committed some minor errors that were recognized and corrected. | Competent performance that indicates working knowledge of ACS but committed a minor error or did not recognize and initiate treatment in a timely manner. | Quickly recognizes and initiates appropriate management of ACS. |
| Communication | 1 | 2 | 3 | 4 | 5 |
|  | Does not communicate well with team. Does not acknowledge team communication. Does not use directed verbal/non-verbal communication. | Weak communication with team. Vague and unclear team communication. Weak directed verbal/non-verbal communication. | Communicates mostly with team, but unclear and vague at times. Usually uses directed verbal/non-verbal communication. | Usually communicates with team, but occasionally is vague or unclear. Usually uses closed-loop communication. Usually uses directed verbal/non-verbal communication. | Exemplary closed-loop communication with team. Consistently uses directed verbal/non-verbal communication. |
| Management of cardiogenic shock | 1 | 2 | 3 | 4 | 5 |
|  | Demonstrates deficient knowledge of cardiogenic shock without recognizing and correcting it. | Weak knowledge of cardiogenic shock. Made errors without recognizing or correcting them. | Competent performance that indicates working knowledge of cardiogenic shock but committed some minor errors that were recognized and corrected. | Competent performance that indicates working knowledge of cardiogenic shock but management could be improved. | Quickly recognizes and initiates appropriate management of cardiogenic shock. |
| Intubation: Organization and selection of supplies | 1 | 2 | 3 | 4 | 5 |
|  | Fails to select correct materials and/or instruments for task. Does not organize appropriately prior to starting task. Does not take into account back up ventilation. | Selects some appropriate materials but forgets others. Unorganized approach. Minimal backup plan. | Slowly selects appropriate materials. Organizes them but not in a manner that helps improve efficiency with performing task or has some materials but not organized before performance of the task begins. | Selects correct materials and instruments for task. Mostly organized. Back-up plan considered. Could improve on time or organization but competent. | Quickly and confidently selects correct materials and instruments for task. Organizes in a deliberate manner that facilitates task. Materials are ready before performance of task begins. Has a back-up ventilation plan ready. |
| Intubation: Correct use of instruments | 1 | 2 | 3 | 4 | 5 |
|  | Student may use an instrument incorrectly and does not correct mistake. | Student may use an instrument incorrectly and may correct mistake or vocalize the error. | Student uses instrument correctly for most part. Occasional errors are made but awareness and correction is demonstrated. | Student holds and uses instruments correctly. Technique mostly smooth, could use some minor suggestions. No errors in use or handling occur. | Student holds and uses instruments correctly throughout using proficient and smooth technique. No errors in use or handling occur. |
